# Comparison of subcutaneous central venous port via jugular and subclavian access in 347 patients at a single center

**DOI:** 10.3892/etm.2012.649

**Published:** 2012-07-30

**Authors:** BILGIN KADRI ARIBAŞ, KEMAL ARDA, ÖZGE ARIBAŞ, NAZAN ÇILEDAĞ, ZEYNEL YOLOĞLU, ELIF AKTAŞ, TURGUT SEBER, ŞEYHMUS KAVAK, YUSUF COŞAR, HIDIR KAYGUSUZ, EKREM TEKIN

**Affiliations:** 1 Department of Radiology, A. Y. Ankara Oncology Education and Research Hospital, Ankara 06200;; 2 Department of Mathematics, Faculty of Science, University of Ankara, Tandoğan, Ankara 06100, Turkey

**Keywords:** central venous port, interventional radiology, cancer

## Abstract

The purpose of the present study was to examine whether patency times, including complications of subcutaneous venous chest port insertion using ultrasonography (US) guidance, differ between jugular and subclavian venous access. Between December 2008 and July 2010, subcutaneous venous chest ports were placed in 347 patients by an experienced team. All single-lumen port catheters were placed into jugular and subclavian veins under US and fluoroscopy guidance. Patency times and complication rates of ports via these routes were compared and the variables were age, gender, access, site of malignancy and coagulation parameters. The success of the jugular and subclavian groups was compared by univariate Kaplan-Meier survival analysis and the multivariable Cox regression test. A total of 15 patients underwent port removal due to complications. As a rate per 100 catheter days, ports were explanted in 7 (0.0092) due to thrombosis, 4 (0.0053) for catheter malposition, one each (0.0013) of port reservoir flip-over, bleeding, port pocket infection, skin necrosis and incision dehiscence, for a total of 15 patients (0.0197). Patency times were not different in the jugular and subclavian veins. Factors were not significant, with the exception of platelet count. There was no significant difference in patency times, including complications, between jugular vein access and subclavian vein access using US. This should be considered when selecting the access method.

## Introduction

Infusion therapy via a subcutaneously implanted venous port system is an attractive alternative to infusion via peripheral veins, peripherally inserted central catheters or tunneled catheters (1). The use of subcutaneous infusion ports has become standard practice to obtain long-term venous access for the administration of chemotherapy, antibiotics or parenteral nutrition (2). First surgically implanted by Niederhuber and colleagues in 1982, subcutaneous venous chest ports were subsequently placed by Morris and coworkers with radiological guidance (3,4). Subcutaneous venous chest ports can be placed into jugular and subclavian veins by landmark and radiological methods. Complication rates have been variously reported depending on approach sites and methods (1–22).

To the best of our knowledge, few studies have been published which compare patency periods of port catheters placed into jugular and subclavian veins using radiological techniques (3). Our objective was to examine whether patency times, including complications of subcutaneous venous chest port insertion using ultrasonography (US) guidance, are different via jugular and subclavian venous access.

## Patients and methods

### Patients and ports

Between December 2008 and July 2010, subcutaneous venous chest ports were placed under US guidance in 347 patients. From December 2008 to July 2009, our guiding principle was to place the ports via the subclavian vein. We subsequently changed our preference to jugular entry due to the greater ease of this technique. The mean age of the patients was 53.8±13.9 years (range, 16–84). Of the patients, 145 (41.8%) were female and 202 (58.2%) were male. All except 1 patient with Behçet's disease had malignancies with or without metastases. The features of the patients and the procedures recorded in this retrospective cohort study were age, gender, access method (jugular or subclavian route), location of primary malignancy, coagulation parameters and complications.

The titanium chambered ports were single-lumen; standard size (7.2 F) port systems with lock mechanisms for catheter attachment. The port type used was Polysite^®^ (Perouse Laboratoires, Ivry-Le-Temple, France). The ports were usually placed on the right side, but were placed on the left if the right side veins had thromboses. The coagulation parameters were tested prior to each port placement (11) and included platelet count, prothrombin time, international normalized ratio (INR) and activated partial thromboplastin time. An effort was made to correct the deficiencies if any coagulopathy was detected. Prophylactic antibiotics were not administered to any patient, including those with fever, until positive culture results. Exclusion criteria were active systemic or local infections, coagulopathy (defined as platelet count <50/nl and/or prothrombin time >18 sec and/or INR>1.5) and the inability to provide informed consent (23).

### Technique of the procedure

Two of the skilled interventional radiologists had 21 years of experience in interventional radiology, including venous catheterization. The other 6 radiologists performed the procedure under their observation. The single-lumen port catheters were placed into the jugular and subclavian veins under US and fluoroscopy guidance in an interventional radiology suite. Patients were placed in the supine position. The pectoral and neck regions were cleansed with povidone-iodine twice. Preprocedural sedation was not administered, except to uncooperative patients. Sterile technique was used, in which the skin at the insertion site was extensively cleansed on the neck or chest. We performed in full surgical scrubs, wearing surgical caps and masks. US guidance (Famio 8; Toshiba, Japan) was performed in all procedures. A high-resolution (11 MHz) linear-array transducer was used with standard accords in all procedures as B-gain, 80 dB; dynamic range, 55; frame per sec (fps), 15 and standard depth, 5.3 cm. Sterile US gel and sterile drapes were used to cover the US probe and cable. A skin incision of 1 cm was made over the jugular or subclavian vein site following local anesthesia administration. Principally, the subclavian vein was punctured in the mid or lateral third of the clavicle to avoid pinch-off. In all patients, venous entry was performed with an 18 G Seldinger needle and the tip of the guide-wire was advanced into the vena cava. After puncture, the subcutaneous pocket was dissected. Following local anesthesia with 2% prilocaine (Citanest^®^; Eczacıbaşı, Turkey), a 2.5- to 3-cm incision was made ∼3 cm caudal to the clavicle with a number 15 bistoury. The pocket site was the same for jugular and subclavian access. Using sharp and blunt dissection, a pocket was created under the fascia of the pectoral muscle and caudally dissected with a clamp and finger. A sponge was placed into the pocket for hemostasis prior to port placement. The catheter connected to the port chamber was tunneled with a trochar to the venous entry site. The catheter was flushed with diluted heparin solution using a Huber needle. The Huber needle remained in the port chamber to prevent flip-over until the end of the procedure. Stay sutures for the port base were not used in any patient, even if the patient had loose subcutaneous tissue. The port catheter was advanced through the peel-away sheath into the vena cava-right atrium junction. After insertion, the catheter tip was evaluated using fluoroscopy. Port efficacy was checked with an aspiration of blood, and the reservoir was flushed with heparinized saline solution (9 cc 0.9% NaCl plus 1 cc heparin) to show any leakage. The incision was closed with resorbable 3-0 vicryl subcutaneous sutures. The skin of the pocket and venous puncture site was closed with 2.0 silk sutures. A digital lung graph was used at the completion of the procedure to evaluate for pneumothorax, catheter kinking and catheter tip position in accordance with the guidelines (23). The port was used for treatment 3 h after the procedure. Port catheters were flushed with heparinized saline solution after each use and, thereafter, monthly if not used.

### Follow-up, analysis of results and statistical analysis

Informed consent was obtained from each patient at the time of the intervention, in accordance with the Helsinki Declaration. All data were obtained from our files and the hospital electronic database system after receiving the permission of the Institutional Review Board (IRB). The follow-up term was from placement until removal of the port, the last follow-up of the patient or mortality date. Patients were censored due to mortality and the last follow-up. Groups were divided by entry methods, which were the jugular and subclavian access groups. Age, gender, access, site of malignancy and coagulation parameters were the variables in the multivariable analysis. The sites of primary malignancies were divided into four regions, which were head-neck, breast-thorax, abdominopelvic and extremity involving >1 region. These regions were grouped together since they were individually small in number.

Complications were defined according to the guidelines of the Society of the Interventional Radiology Standards of Practice Committee (5). Major complications result in admission to a hospital for therapy (for outpatient procedures), an unplanned increase in the level of care, prolonged hospitalization, permanent adverse sequelae or mortality (5). Complications were divided into early (<30 days) and late (>30 days) as in the reporting standards (23).

Event (failure) was defined as unplanned port removal due to complications, so the groups were divided into failure and success. Patency times of the ports via these routes were compared using univariate Kaplan-Meier survival analysis and the multivariable Cox regression test. P<0.05 and 95% confidence interval (CI) in the analysis were considered to indicate a statistically significant difference. Survival plots for catheter patency were obtained from the statistical analysis. Statistical analysis was performed by a mathematician (Ö.A.).

## Results

Nine patients, of which 1 was in the subclavian group, had abnormal bleeding parameters; these were corrected before their ports were implanted. No major complications were detected during the procedure. Two major complications occurred following the procedure in the jugular group; a late complication after 132 days in a 69-year-old male patient, who experienced infection by skin necrosis, and an early complication on the second day, which was bleeding due to prolonged INR (1.70) in a 29-year-old male patient to whom blood was administered before the port was removed. Patient features and patency periods of subclavian and jugular catheters are shown in Table I. Port catheters were placed into jugular and subclavian veins in 248 (71.5%) and 99 patients (28.5%), respectively. The mean number of catheter days was larger in the subclavian than in the jugular access groups, 270.4 vs. 199.2 days. Ports were placed on the left side in 4 patients; jugular in 2 and subclavian in the remaining 2 patients.

Complications without port removal were observed in 4.9% of patients (17 patients). These included erythema and itching in 2 patients, opening of sutures in 3 patients and bleeding in 12 patients. Ports were explanted due to the end of treatment in 5 patients and 20 mortalities were observed during follow-up, which were censored. Port removal due to complications was observed in 15 patients, 9 in the jugular group and 6 in the subclavian group (Table II).

**Figure 1:**
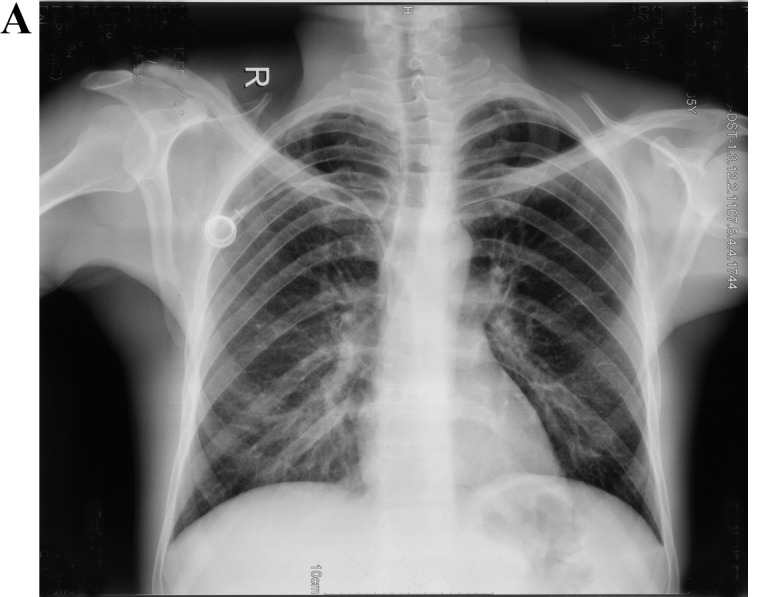

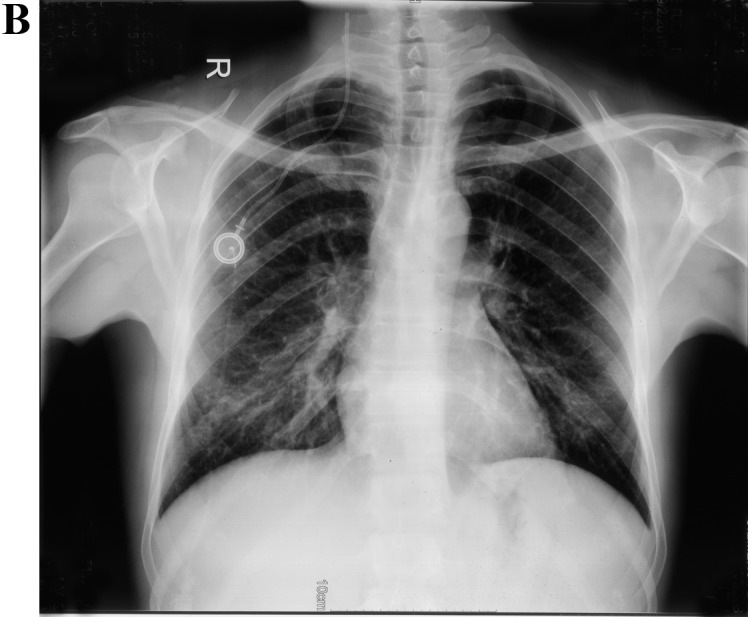
A 36-year-old male patient with colon carcinoma. (A) Upright posteroanterior chest radiograph showing that a right subclavian port was implanted into the chest wall and that the catheter was placed into the lower half of the vena cava just after the procedure. (B) Malposition due to dislocation of this port catheter can be observed in the right jugular vein. This developed 3 months later. The catheter was subsequently placed back into the vena cava via jugular access.

Thrombosis occurred in 2 patients, malposition in 3 (Fig. 1) and infection in 1 patient when subclavian access was used, whereas there was thrombosis in 5 patients (3 catheter thromboses, 1 jugular and the other brachiocephalic vein thrombosis) and 1 case each of malposition, bleeding, reservoir flip-over and skin necrosis with wound infection in the jugular access group. Of the complications, 6 were early (4 in the jugular and 2 in the subclavian group) and 9 were late (5 in the jugular and 4 in the subclavian group).

The port removal rate due to complication per 100 port catheter days was 0.00182 in the jugular and 0.00224 in the subclavian entry groups. Also, the port infection rate per 100 port catheter days regarding the procedure was 0.0020 in the jugular vs. 0.0037 in the subclavian group.

**Figure 2:**
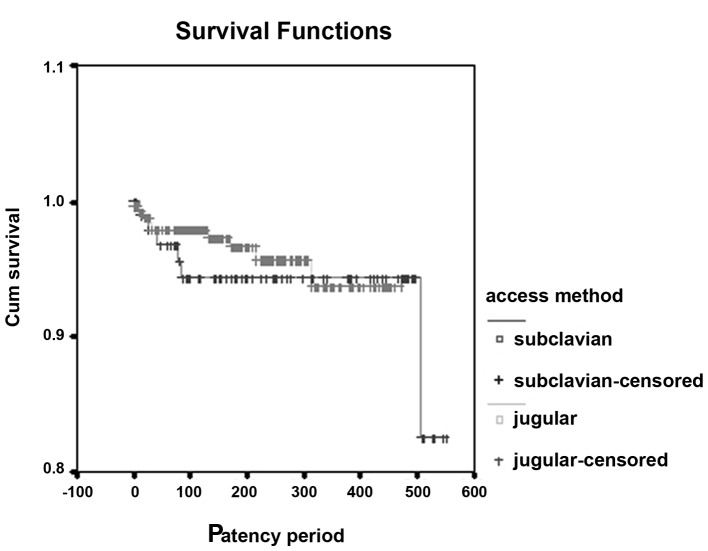
Graph of Kaplan-Meier survival test showing the cumulative catheter patency times of the jugular and subclavian vein access groups with no significant differences.

The graph in Fig. 2 shows the cumulative survival for catheter patency times of the jugular and subclavian access groups in the Kaplan-Meier survival analysis. Log-rank test did not detect any significant differences between the groups (P=0.662).

**Figure 3:**
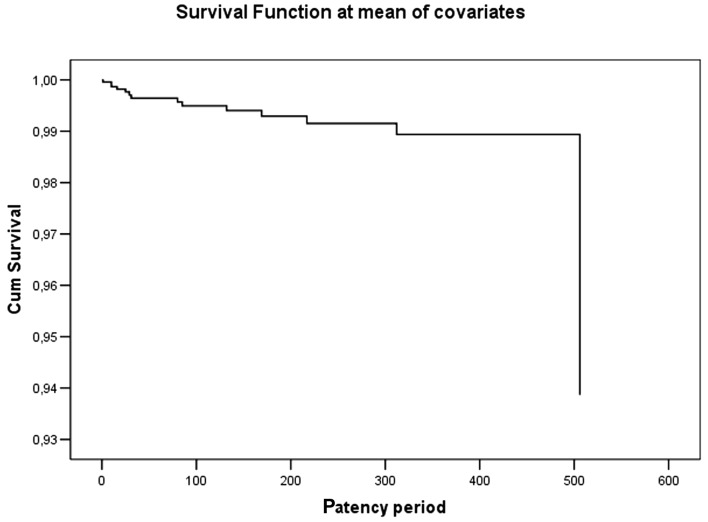
Graph of Cox multivariable regression test showing mean catheter patency time. Confidence interval (CI) for exponent B was 95%, entry 5% and removal 10%, maximum iterations 20, model entry and model display information were selected at each step.

The graph in Fig. 3 shows the total cumulative survival for mean catheter patency time by the multivariable Cox regression test. Age (P=0.252), gender (P=0.775), access vein groups (P=0.369), site of primary malignancy (P=0.607) and coagulation parameters, with the exception of platelet count (P=0.043), were not significant variables in this multivariable test. Coagulation parameters were platelet count (P=0.043), prothrombin time (P=0.526), INR (P=0.289) and activated partial thromboplastin time (P=0.087) in the multivariable test. Their platelet counts (/nl) were 275.5±96.8 in the failure group and 309.4±121.0 in the successful group.

## Discussion

Image-guided insertion of subcutaneous chest ports has a number of advantages compared with unguided insertion, resulting in a higher success rate and fewer complications (6). The major difference between the techniques is the use of fluoroscopy and US (7,8). US guidance reduces the number of mechanical complications, the number of catheter placement failures and the time required for insertion. However, US guidance use during subclavian venous catheterization has had mixed results in clinical trials, probably due to anatomical reasons (9).

Our complication rate was low due to the use of imaging guidance, compared with the complication rates of the landmark method, as reported in the literature. The use of two-dimensional ultrasound (2D-US) guidance during internal jugular catheterization has been demonstrated to lead to a reduction in the rates of unsuccessful cannulation, carotid artery puncture and hematoma formation when compared with the anatomical landmark technique (10–13).

In the literature, the most common complications after implantation were thrombosis, catheter dysfunction and infections (2). Catheter-related thrombosis is one of the most significant complications; its frequency ranges from 0.67 to 5% (2). Our thrombosis rate was within this range at 2% (0.0092 per 100 catheter days) and was slightly higher in the jugular access group. However, it is debatable whether Plumhans *et al* (3) observed vein thrombosis in 3% of the subclavian group and in 1% of the jugular group when thrombosis was reported in 1% of subclavian ports (8) and in 1.7% of jugular placements (6) in other studies.

Female patients and patients with lung cancer also had an elevated risk of developing a thrombosis (2). We detected more frequent thrombosis in female patients (71.4% compared with 28.6% in male patients) but this was not significant in multivariable analysis. Also, we were unable to differentiate thrombosis detection between patients with various malignancy localizations.

The risk of catheter-related infection was reportedly lower for subclavian vein access than for jugular or other access sites; however, no randomized trial has satisfactorily compared infection rates for catheters placed in jugular, subclavian and femoral sites (9,11,13–15). We could neither find a difference in the infection rates (0.0020 in jugular vs. 0.0037 in subclavian per 100 catheter days) nor incubate the responsible microorganism in cultures of the two port site infections due to the antibiotic treatments administered.

Catheter-related complications also include necrosis of the skin, malpositioning of the catheter tip, dislocation, embolization, rupture and compression of the catheter, although these are rare (2). Catheter tip position is less susceptible to migration when placed through the internal jugular vein (3). Accordingly, we detected this in the present study, and our catheter malposition rate per 100 catheter days was 0.0020 in the jugular vs. 0.0112 in the subclavian route. Skin necrosis may be observed in port placement (2,4). Skin erosion has been reported in 0 to 1% of cases in the literature (1,4), and our rate was 0.3% (0.0013 per 100 catheter days).

Subclavian venipuncture has been the most popular route for transition and long-term central venous cannulation, although perioperative complications occur in up to 12% of the patients (11). Currently, radiologists prefer the internal jugular vein since it is makes catheterization easier (3,4,6,13). The main advantages of jugular versus subclavian access are the reduced periprocedural complications, better ultrasound control, no pinch-off and lower migration and venous stenosis rate (3,10,16). Also, Plumhans *et al* (3) reported that their results demonstrated an approximately 50% reduction of pain perception when the port-catheter was introduced via the internal jugular vein. Conversely, Lorch *et al* (1) preferred access through the subclavian vein since the distance to the vena cava and right atrium is short so no tunneling is necessary, thus shortening the procedure time; it also requires no second incision at the neck, which may be an advantage, especially in cachectic patients.

When possible, a lateral puncture of the subclavian vein should be performed if subclavian access is chosen to avoid pinch-off (1,14,15). Fluoroscopic and/or ultrasound-guided access to the subclavian vein also prevents catheter buckling or breakage due to ‘pinching’ between the first rib and the clavicle (17). The success rate of the technique was higher in subclavian access in 55 patients for Brooks *et al* (18) with US guidance. Also, we did not observe visible catheter pinching, but some pinch-off may have an effect on catheter thrombosis in subclavian entry.

Previously, two studies (14,15) revealed that port placement via the subclavian vein was as successful as studies performed via the jugular vein reported in the literature. Biffi *et al* (7) prospectively compared subclavian and jugular port placements using the radiological and landmark methods and found no differences in success. Furthermore, we did not find any significant differences between catheter patency periods of subclavian and jugular port placements, including complications, using the same method.

Port inversion (turning over inside the port's fibrous capsule) is an extremely rare complication (19,20). Postulated risk factors for port inversion include loose or redundant subcutaneous tissues and large pocket size (19). Thus, fixing the port chamber in the subcutaneous tissue with sutures is not necessary if the port pocket size is adequate (1). Also, certain studies have, as we observed in the present study, demonstrated that starting chemotherapy on the day of port catheter implantation is safe, and does not increase the frequency of acute or chronic complications (21).

This study has some restrictions. Among them, the number of the patients was limited due to single center experience. Also, the patients were not equally distributed between the two access groups whereby the mean follow-up period of the subclavian group, whose success would be expected at a lower rate, was longer than that of the jugular group.

In our presented data, the periprocedural complication rate was 0%. We would like to emphasize that skilled interventional staff performed the procedure. Our experience has an important role in the absence of periprocedural complication, especially in subclavian entry. Thus, our choice of entry method had a minimal effect on technique success.

We did not find any factor to be effective on catheter patency times by multivariable analysis, with the exception of platelet count. However, the difference in platelet counts was approximately P=0.05. This may be due to the limitations of our study. This may be examined in further studies.

The rate of symptomatic thrombosis in subclavian access was higher in the study by Trerotola *et al* (22). However, US markedly decreased failure and complication rates in subclavian entry in the study by Brooks *et al* (18), which was also true in our study. Data extracted from the current study may increase the amount of evidence in the literature for the positive effect of US in subclavian entry.

In conclusion, there was no significant difference in patency times, including complications, between jugular vein and subclavian vein access when using US. This should be considered when selecting the access method.

## Figures and Tables

**Table I:** Demographic features of patients.

Characteristics	Total	Jugular group	Subclavian group
Age (years)^a^	53.8±13.9	53.0±14.0	55.7±13.7
Gender^b^			
Female	145 (41.8)	110 (44.4)	35 (35.4)
Male	202 (58.2)	138 (55.6)	64 (64.6)
Access vein^b^	347 (100)	248 (71.5)	99 (28.5)
Platelet count/nl^a^	307.9±120.2	306.9±123.9	310.4±110.8
Prothrombin time (sec)^a^	11.8±1.5	11.4±1.2	12.8±1.7
International normalized ratio (INR)^a^	0.980±0.137	0.946±0.111	1.068±0.157
Activated partial thromboplastin time (sec)^a^	28.2±4.5	28.1±4.5	28.3±4.7
Localization of primary malignancies^b^			
Head-neck	62 (17.9)	45 (18.2)	17 (17.2)
Breast-thorax	29 (8.4)	21 (8.5)	8 (8.1)
Abdominopelvic	237 (68.3)	170 (68.8)	67 (67.7)
Extremity-other^c^	18 (5.2)	11 (4.5)	7 (7.1)
Mortality^b^			
Positive	20 (5.8)	12 (4.8)	8 (8.1)
Negative	327 (94.2)	236 (95.2)	91 (91.9)
Patency periods (days)^d^	219.5±145.0 (1–550)	199.2±122.5 (1–471)	270.4±180.9 (1–550)

a Mean ± 2SD;

b total of malignancies = 346, n (%);

c three patients had malignancies involving 2 regions;

d mean ± 2SD (range).

**Table II:** Complications leading to port removal.

		Jugular		Subclavian			
Complication	n (%)^a^	n (%)	P-value	n (%)	P-value	Term (days)	Rate/100 catheter days
Thrombosis^b^	7 (2.0)	5 (2.0)	0.0101	2 (2.0)	0.0074	375	0.0092
Malposition	4 (1.2)	1 (0.4)	0.0020	3 (3.0)	0.0112	833	0.0053
Flip-over	1 (0.3)	1 (0.4)	0.0020	0	0	312	0.0013
Hemorrhage	1 (0.3)	1 (0.4)	0.0020	0	0	1	0.0013
Infection^c^	2 (0.6)	1 (0.4)	0.0020	1 (1.0)	0.0037	142	0.0026
Skin necrosis^c^	1 (0.3)	1 (0.4)	0.0020	0	0	132	0.0013
Total^d^	16 (4.4)	10 (4.0)	0.0182	6 (6.1)	0.0224	1,795	0.0197

Total catheter patency days 76,170 in total, 49,405 in jugular and 26,765 in subclavian routes.

a Patency rates were 96.4% in the jugular access and 93.9% in subclavian access groups.

b Five (71.4%) female patients and 2 (28.6%) male patients.

c Infection accompanied by skin necrosis in 1 patient.

d Sixteen complications were observed in 15 patients.
